# Skin and Muscle Closure Techniques Following Large-Scale Osteosarcoma Removal: A Comparative Analysis

**DOI:** 10.7759/cureus.64258

**Published:** 2024-07-10

**Authors:** Christopher R Meretsky, Brandon Krumbach, Jay Popovich, Mohammed Ajebli, Anthony T Schiuma

**Affiliations:** 1 Surgery, St. George's University School of Medicine, Great River, USA; 2 Anatomy, St. George's University, Great River, USA; 3 Internal Medicine, St. George's University School of Medicine, Great River, USA; 4 Biology Sciences, Moulay Ismail University, Faculty of Sciences and Technologies, Errachidia, MAR; 5 Orthopedic Surgery, Holy Cross Hospital, Fort Lauderdale, USA

**Keywords:** wound closure, flap reconstruction, negative-pressure wound therapy, osteosarcoma, skin and muscle closure

## Abstract

Osteosarcoma (OS), the most prevalent form of bone cancer, typically arises in osteoblast cells responsible for generating new bone. The bone produced by these cancer cells is weaker compared to healthy bone. OS is an aggressive bone cancer that often requires extensive resection, leaving behind substantial soft tissue defects. Successful closure after tumor excision is critical for wound healing and postoperative recovery. However, the optimal approach varies depending on factors like defect size and location. After extensive resection of OS, restoring the integrity of the affected area demands careful closure of both the skin and underlying muscle. The appropriate closure technique depends on the size and location of the soft tissue defect. The main objective of this systematic review is to evaluate and compare different surgical techniques for closing skin and muscle layers following large-scale OS removal. Through a systematic review methodology, we conducted an extensive analysis of the existing body of literature on this topic, drawing from relevant research papers published over the past two decades. This allowed us to collectively evaluate and synthesize available data on the subject. This review found that negative pressure wound therapy (NPWT) and flap reconstruction are the main surgical approaches used to close skin and muscle following extensive OS resection, which commonly results in large soft tissue defects due to the nature of tumor removal. Furthermore, NPWT was the most widely used method for closing soft tissue defects after major OS removal, while flap reconstruction was also common when NPWT was not appropriate or the defect was too large. An integrated approach combining vacuum therapy, skin stretching, and occasional flaps seeks to primarily close large defects after OS resection through optimized healing and tension reduction to achieve the best postoperative results.

## Introduction and background

Osteosarcoma (OS), the most prevalent form of bone cancer, typically arises in osteoblast cells responsible for generating new bone. The bone produced by these cancer cells is weaker compared to healthy bone [1]. OS occurrs globally at a rate of 3.4 cases per million individuals on an annual basis [2]. According to the American Cancer Society (ACS), OS is considered a rare form of cancer. Approximately 1,000 new cases are identified in the United States each year, with around half of these occurring in children and teenagers. The majority of OSs manifest in individuals aged between 10 and 30, with teenagers being the most impacted age group; however, OS can develop in individuals of any age. While the majority of cases occur in younger age groups, about one in 10 cases are found in individuals over 60. OSs constitute around 2% of childhood cancers, but they represent a significantly lower proportion of adult cancer cases [3]. OS typically develops in the metaphyseal region at the ends of long bones and often spreads into the epiphysis [4,5]. The development of OS is believed to be triggered by a cancer-causing event in the precursor cells of osteoblasts, which are dividing quickly during the growth of the skeleton [6]. The existing standard treatment, which combines surgery and chemotherapy, results in about 60% [7] of patients with localized disease in the limbs achieving long-term survival without the disease recurring. However, for patients with primary metastases or tumors in the axial skeleton, the long-term, disease-free survival rate drops between 20% and 30% [8,9].

Following extensive OS removal, restoring the integrity of the affected area requires careful attention to both skin and muscle closure. Techniques vary depending on the size and location of the defect but generally involve either primary closure, if the tissue edges can be approximated, or more complex methods like skin grafts or muscle flaps for larger defects [10]. Additionally, the use of robust flaps for soft tissue cover is recommended, especially when primary closure is not possible [11]. This approach helps to prevent tension on the incisions, which can lead to prolonged delays in wound healing and negatively impact the delivery of adjuvant treatments. Furthermore, the use of acellular dermis reconstruction with skin graft and vacuum-assisted closure (VAC) devices can provide excellent coverage alternatives and promote wound healing in high-risk patients [6].

The objective of this systematic review is to evaluate and compare different surgical techniques for closing skin and muscle layers following large-scale OS removal. Furthermore, this review aims to assess the outcomes and effectiveness of two main strategies: negative pressure wound therapy (NPWT) and reconstructive flap procedures. By analyzing relevant studies that utilized these closure methods, the goal was to determine their relative benefits, limitations, and appropriateness for different defect presentations. Identifying the most effective techniques could help guide clinical decision-making and the establishment of best practice guidelines for reconstructing large defects post-OS excision. The comparative analysis sought to provide evidence to support the selection of the closure option most likely to result in primary healing and restoration of form and function.

## Review

Methods

Study Selection

**Figure 1:**
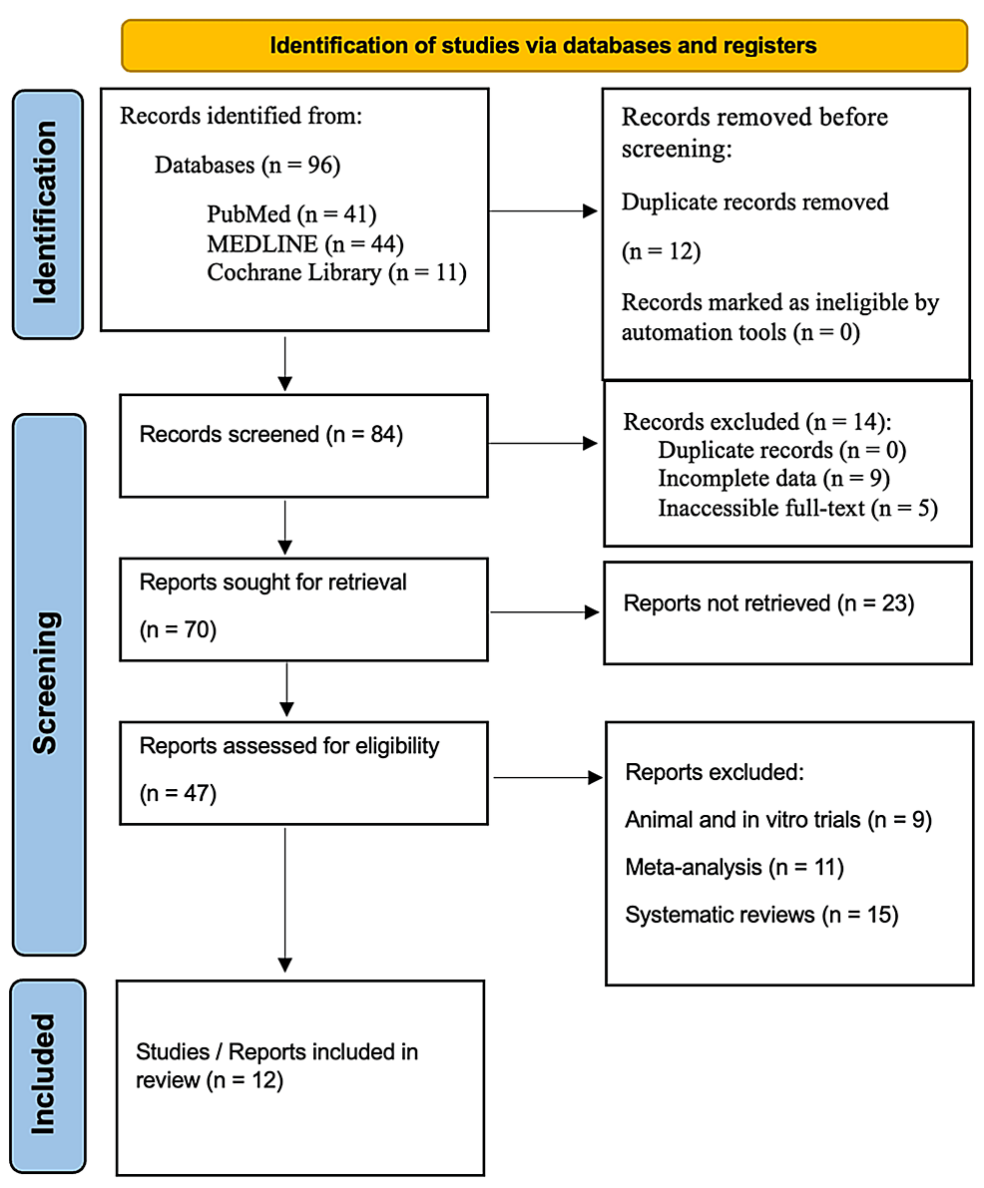
PRISMA flowchart: literature search and study selection n, number; PRISMA, Preferred Reporting Items for Systematic Reviews and Meta-Analyses Ref. [12]

In accordance with the Preferred Reporting Items for Systematic Reviews and Meta-Analyses (PRISMA) guidelines, a systematic review was undertaken. The databases of PubMed, MEDLINE, and the Cochrane Library were thoroughly searched for studies that were published in the span of two decades, from 2004 to 2024. The search was conducted using specific keywords, such as “sarcoma removal,” “wound closure,” “flap reconstruction,” “negative pressure wound therapy,” “postoperative infection,” and “postoperative pain.” The PRISMA guidelines were followed to ensure transparency and reproducibility in the review process (Figure 1).

Inclusion Criteria

The studies that were considered for inclusion in this review had to meet certain criteria. Firstly, they had to involve human subjects who were undergoing large-scale sarcoma removal. Secondly, they needed to compare different techniques used for wound closure. Thirdly, they had to report outcomes on various factors, such as the length of hospital stay, the level of postoperative pain experienced by the patients, and the rates of postoperative infection. Lastly, the studies had to be published in English.

Exclusion Criteria

However, we did exclude some studies from our selection. Studies that did not report adequate data specifically on scale OS removal cases were excluded. We also did not include meta-analyses, reviews, or editorials that lacked original findings. Research exclusively conducted in animal models was also not considered. This discerning selection process served to reinforce the relevance and reliability of our review by focusing only on primary studies directly related to the human population of interest.

Data Extraction

Once the studies were selected based on the inclusion criteria, data were extracted from them. The extracted data included information on the design of the study, the size of the sample used, the demographics of the patients involved, the type of sarcoma that was removed, the techniques used for closing the wound, and the outcomes that were reported in the study. This comprehensive data extraction allowed for a thorough and detailed analysis of the studies.

Results

Negative Pressure Wound Therapy

A systematic process was undertaken to identify relevant studies for the present review. Multiple databases were searched using predefined search criteria related to wound closure techniques following extensive OS resection. The titles and abstracts of retrieved articles were screened for eligibility based on inclusion/exclusion criteria. Key factors considered included the utilization of pressure wound therapy or flap reconstruction methods, human subjects, full-text availability, and publication in the English language. After a thorough screening, 12 studies met the inclusion criteria and were fully reviewed. Of these, six studies specifically investigated the use of various NPWT techniques. The other six reviewed different flap reconstruction approaches for closing large soft tissue defects. Both modalities represented valuable options for the restoration of integrity following significant OS removal. By selecting an appropriate sample of high-quality studies representing these two main closure methodologies, this systematic review aimed to offer meaningful insights into optimal post-resection wound management.

Table 1 comprehensively synthesizes the current understanding of the application and efficacy of NPWT in the management of OS. A thorough search of the clinical trial, observational, and case study literature published within the past two decades was conducted. Details from relevant publications were extracted and analyzed, focusing on study design elements, such as sample sizes, comparison groups, NPWT application durations, monitored side effects, employed outcome measures, and reported conclusions. These elements were then assimilated into Table 1 to provide an overview of NPWT techniques used, along with observed safety profiles and potential benefits. A total of 12 studies met the inclusion criteria and were reviewed. Various NPWT protocols, wound closure adjuncts, and treatment settings were identified. Primary outcomes centered around wound healing times, complication rates, postoperative infections, and the need for additional procedures. Earlier case studies provided a preliminary signal of NPWT’s safety and efficacy. Larger trials subsequently evaluated discrete protocol optimizations. This structured synthesis offers clinicians and researchers a comprehensive yet succinct perspective on the evolving understanding of NPWT applications for OS, highlighting knowledge gaps that could guide future investigations.

**Table 1:** Summary of clinical studies on NPWT techniques following large-scale osteosarcoma removal (2004-2024) NPWT, negative pressure wound therapy

Reference	Study type	Sample size and treatment groups	Technique	Period of application	Success of the 1sttechnique	Success of the 2nd technique	Side effects	Main outcome	Main conclusion
Kim et al. (2016) [13]	Observational study	9 patients	NPWT and latissimus dorsi myocutaneous flap (LDMF)	14 months	The defects ranged in size from 14 × 8 cm to 18 × 15 cm	The flap size ranged from 15 × 10 cm to 18 × 15 cm	All the flaps had a smooth recovery, with the exception of one patient who suffered from persistent seroma and wound separation.	During the follow-up periods, which averaged 14 ± 6.1 months, there were no instances of osteomyelitis reoccurring.	Given the outcomes from this continuous patient series, we propose that this technique could offer a different strategy for managing severe osteoradionecrosis in the gluteal area.
Yuan et al. (2023) [14]	Randomized controlled trial	138 patients received flap reconstruction: 37 underwent free flap reconstructions were not included in the study. The remaining 101 patients were categorized into two groups: 51 in the NPWT group, and the control group consisting of 50 patients.	NPWT and control group	4-7 days	Both groups had similar patient demographics and patient and wound risk factors for impaired wound healing.	Both groups had similar patient demographics and patient and wound risk factors for impaired wound healing.	None reported	No significant differences were found in flap necrosis, surgical-site infection (SSI), wound dehiscence, hospital stay duration, and functional recovery rates. Cost analysis suggests that using NPWT instead of flaps for the initial seven days after surgery could be a more cost-effective option in our environment.	Using NPWT over flaps postoperatively was safe and effective, with benefits including better exudate control, reduced edema, and potential cost savings.
Gigliotti et al. (2023) [15]	Case report	A 56-year-old female with a history of meningioma status post subtotal resection and extensive radiation therapy presented with osteoradionecrosis (ORN).	NPWT	2 weeks	2 × 1.5 cm on the left parietal aspect of the flap healing is secondary intent	-	Injury to the hypoglossal nerve, separation of the flap insertion, and reopening of the neck access incision necessitating further surgical intervention.	NPWT effectively treats open calvarial wounds caused by osteoradionecrosis, leading to improved flap survival and successful reconstruction. The patient's flap showed 100% viability, and there was a 2 × 1.5 cm area on the left parietal aspect of the flap that was healing via secondary intention.	We show that NPWT effectively manages open calvarial wounds caused by osteoradionecrosis.
Stumpfe et al. (2020) [16]	Case report	2 cases of pseudotumors: 12 and 29 years after latissimus dorsi (LD) transfer	NPWT	Several days	-	-	None reported	The case report detailed two occurrences of uncommon late-onset pseudotumors that manifested many years following the initial LD reconstruction.	In rare instances of late-onset pseudotumors at the latissimus dorsi muscle flap donor site, the application of NPWT with instillation and dwell time has been shown to effectively decrease wound size and lower shear forces.
Zhang et al. (2016) [17]	Observational study	9 patients with submandibular fistulas after reconstruction for osteoradionecrosis treated with NPWT	NPWT	18 months	-	-	None reported	The fistulas demonstrated exceptional healing and did not show any signs of recurrence or infection.	NPWT is a safe and effective method for treating submandibular fistulas following reconstruction for osteoradionecrosis.
Sakellariou et al. (2011) [18]	Observational study	32 patients treated for bone and soft tissue sarcomas and secondary wound-healing complications	NPWT	15 to 30 days	-	-	None reported	A significant statistical variance was observed in the duration of hospital stay between the conventional wound treatment group and the NPWT group.	Utilizing NPWT to address complex wound healing in sarcoma patient’s post-tumor surgery proves to be safe and efficient. This approach is linked to reduced overall complication rates, lower infection occurrence, decreased necessity for additional surgeries, and a lower total expense for wound healing treatment.

This systematic review provides a comprehensive overview of the current understanding and efficacy of NPWT in the management of OS. A thorough analysis of clinical trials, observational studies, and case reports published within the past two decades was conducted. Key details from relevant studies, including sample sizes, treatment protocols, outcomes assessed, and conclusions, were extracted and summarized concisely in Table 1. The analysis reveals that NPWT demonstrates promise across several domains. It appears to offer an alternative treatment strategy for severe osteoradionecrosis, particularly in challenging anatomical sites like the gluteal region. The application of NPWT following flap procedures yielded improved wound healing outcomes, such as reduced edema and exudate. Additionally, NPWT exhibited efficacy in managing open calvarial wounds caused by osteoradionecrosis, improving flap survival and reconstruction success. When applied to address complex post-surgical wound challenges in sarcoma patients, NPWT contributed to lower complication rates, fewer wound infections, reduced re-interventions, and potential cost savings. While early case reports provided preliminary evidence of NPWT’s safety profile, larger subsequent trials investigated optimized protocols and applications. This review underscores the evolving, yet still developing, status of NPWT within OS treatment. It highlights the need for additional research to establish optimal protocols, address existing knowledge gaps, and develop clear clinical guidelines for NPWT’s safe and effective implementation.

Flap Reconstruction Techniques

Table 2 provides a comprehensive overview of clinical studies examining flap reconstruction techniques following extensive OS resections between 2004 and 2024. The studies include a diverse range of patient demographics and disease characteristics, providing insights into the applicability of these approaches across different OS cases. Specifically, the table outlines the reconstruction methods employed, such as the use of vascularized fibula flaps and osteocutaneous radial forearm flaps, as well as combination techniques, detailing the technical aspects of the procedures.

Adjunct techniques utilized in conjunction with the flap reconstructions are also described, such as virtual surgical planning, patient-specific cutting guides, and NPWT, highlighting the evolving multimodal nature of the interventions. The sequencing and timing of the reconstructive surgeries relative to the initial OS resection are documented, providing perspective on the optimal staging of these complex procedures.

Key outcomes like rates of hardware removal, wound healing, functional restoration, and oncologic control are summarized to assess the efficacy and safety of the flap reconstruction approaches. Finally, the table synthesizes the overarching findings and conclusions from the studies to distill the current state of evidence, identifying areas for further refinement of these techniques in OS management shown in Table 2.

**Table 2:** Summary of clinical studies on flap reconstructions techniques following large-scale osteosarcoma removal (2004-2024)

Reference	Study design	Patients characteristics	Surgical details	Technique	Timeframe	Outcome	Conclusion
Chana et al. (2004) [19]	Case report	13 patients	Segmental mandibulectomy, immediate reconstruction with free fibula flap, and simultaneous placement of osseointegrated implants	Segmental mandibulectomy and immediate free fibula osteoseptocutaneous flap reconstruction with endosteal implants	40 months (range, 18-70 months)	Successful reconstruction, no re-explorations or partial flap losses	Reduced risk of recurrence, reliable mandibular reconstruction, and fewer surgical procedures
Lazarides et al. (2014) [20]	Case report	A 25-year-old woman presented for a second opinion regarding treatment of a mandibular chondroblastic osteosarcoma	Prior to surgery, a virtual surgical planning session was conducted. A stereolithographic model of the mandible was also created to perform a customized 2.4 mm titanium reconstruction plate from Stryker CMF.	Vascularized osteoseptocutaneous fibula flap	1 month	Thirdly, a vascularized osteoseptocutaneous fibula flap offers a robust and versatile medium for a custom template and repair of the mandible. Utilizing this type of free flap provides a viable tissue source that can withstand the stresses of chemotherapy.	Nevertheless, advancements in modeling and template usage have simplified the attainment of both aesthetically pleasing and functionally effective outcomes.
Bidra et al. (2009) [21]	Clinical report	Rehabilitation of the maxillofacial region for a 7-year-old boy who had a significant portion of his left mandible removed during treatment for osteosarcoma.	After surgically removing the left half of the mandible from the angle to the parasymphyseal region, a free flap consisting of bone and skin from the fibula was employed to effectively rebuild the mandible.	Free fibula flap and implant-supported prosthesis	2 weeks	The prosthesis helped to improve the patient's appearance, functionality, and psychological well-being. The significance of utilizing a variety of specialists and careful treatment planning for a child with a reconstructed jaw was emphasized.	Due to the risk of this disease recurring locally and the patient's ongoing facial development, a multidisciplinary team is closely monitoring their condition.
Ghanem et al. (2016) [22]	Case report	A 18-year-old male with metastatic osteosarcoma of the mandible	Radical composite salvage surgery was conducted, involving a near-total mandibulectomy, total oral glossectomy, bilateral buccal mucosa excision, and excision of the entire lower lip, submental, and upper central neck skin.	Multiple flap reconstruction	3 years	Reconstruction of extensive facial defects can be effectively achieved using a combination of two free flaps: an osteocutaneous fibular flap and a five-component subscapular artery-based flap. This dual flap approach consistently yields favorable outcomes.	Reconstructive surgeons should anticipate the possibility of future procedures to enhance the initial reconstruction. In such cases, preserving the internal mammary vessels and the cephalic vein provides valuable options for subsequent procedures.
Brauner et al. (2017) [23]	Case report	A 25-year-old male diagnosed with chondroblastic osteosarcoma of the pre-maxilla	The case study aimed to rehabilitate the patient through an implant-supported prosthesis comprising three distinct components: a titanium base screwed onto the implants, a titanium primary structure assembled on the base, and a composite-coated secondary structure that replicated the teeth and gums. During the surgical procedure, the clinicians placed 6 dental implants.	Fibula free flap reconstruction	-	The goal of implant rehabilitation is to enhance the quality of life for these patients by enabling proper retention of removable prostheses and reducing the load placed on vulnerable soft tissues.	In modern prosthodontics, prosthetically guided rehabilitation has become the primary rehabilitation protocol, particularly for oncology patients who have experienced significant tissue loss and anatomical changes.
Lese et al. (2023) [24]	Observational study	202 patients undergoing sarcoma resection over the course of 5 years	Individuals who underwent flap reconstruction procedures following the removal of sarcoma tumors between January 2014 and December 2018	Flap reconstruction following surgical resection of soft tissue and bone sarcoma in the setting of (neo)adjuvant therapy	5 years	Postoperative complications were observed in 37.7% of the patients, and the flap failure rate was 4.4%. Preoperative chemotherapy was significantly associated with a higher occurrence of early infection and late wound dehiscence. Intraoperative radiotherapy was found to be associated with the development of late seromas and lymphedema.	Reconstructive procedures involving pedicled or free flaps can be a reliable approach, but they can also be challenging in the context of sarcoma surgery. Higher rates of postoperative complications are anticipated in cases where neoadjuvant therapy has been administered, as well as in the presence of certain comorbid conditions.

Table 2 conducts a thorough review of clinical and case studies from the past 20 years focusing on the application of flap reconstructions following extensive OS resections. The review reveals several noteworthy findings, including a decreased risk of tumor recurrence, reliable reconstruction of the mandible, and a reduced need for multiple surgeries. Notably, the vascularized osteoseptocutaneous fibula flap has emerged as a highly effective solution for these complex cases. This flap provides durable, adaptable tissue that can withstand chemotherapy treatments, facilitating the creation of customized implant templates and successful mandibular repairs. Incorporating prosthetics with this technique has proven invaluable in enhancing patients’ aesthetics, functionality, and psychological well-being. The studies emphasize the importance of interdisciplinary collaboration and meticulous treatment planning, particularly for pediatric patients requiring jaw reconstruction. A noteworthy approach highlighted is the combination of two free flap procedures: an osteocutaneous fibular flap along with a five-component subscapular artery-based flap, which consistently yields positive outcomes in achieving effective facial defect reconstruction.

Overall, the review underscores the major advancements made in flap reconstructions for extensive OS resections over the past two decades. Specifically, utilizing vascularized flaps like the osteoseptocutaneous fibula flap and incorporating prosthetics have contributed to improved clinical outcomes, lower recurrence risks, and enhanced functional and aesthetic results for patients. The interdisciplinary approach and careful preoperative planning are also identified as critical factors for success with these complex reconstructive procedures.

Discussion

Recent research demonstrated that the current approach to OS treatment involves surgical removal of all visible diseases, combined with systemic chemotherapy to manage micro-metastatic disease. This strategy results in a five-year event-free survival (EFS) rate of around 70% for patients with localized OS. However, patients with metastatic or recurrent disease have significantly worse outcomes, with survival rates below 20% [25].

Saving the limb is the standard approach for managing primary bone tumors. However, limb-sparing procedures often have high rates of wound issues, especially for tumors in the lower extremities [26]. As such, it is important to identify and utilize interventions that can help reduce the chances of wound complications following limb-preserving removal of lower extremity bone tumors [27]. Interestingly, minimizing postoperative wound risks in these complex surgeries is crucial.

This systematic review reveals that NPWT and flap reconstruction techniques are primarily utilized by surgeons to close skin and muscle following the removal of large-scale OSs. Evidently, OS, a type of bone cancer, commonly necessitates extensive surgical resection to excise affected bone. Removal of a large OS often results in a substantial wound that poses a significant reconstructive challenge. Interestingly, the review found NPWT and flap reconstruction to be the predominant closure approaches employed. NPWT, also called VAC, uses negative pressure to promote wound healing. Flap reconstruction involves the use of vascularized skin, muscle, or other tissues to cover and close the defect. In the same context, studies demonstrate that these techniques effectively facilitate wound healing and closure post-resection of extensive OSs. This allows patients to recover from surgery and potentially receive adjuvant therapies as needed.

The systematic review highlights the importance of specialized closure methods like NPWT and flap reconstruction in managing complex wounds resulting from surgical OS treatment. The findings offer useful insights for healthcare professionals guiding care for patients with large bone tumors.

Negative Pressure Wound Therapy

This systematic review provides a comprehensive overview of the current understanding and efficacy of NPWT in managing OS. A thorough analysis was conducted of clinical trials, observational studies, and case reports published in the last two decades. Table 1 concisely extracts and summarizes key details from relevant studies, such as sample sizes, treatment protocols, outcomes assessed, and conclusions. The analysis reveals that NPWT shows promise in several areas. It appears to offer an alternative treatment strategy for severe osteoradionecrosis, particularly in challenging anatomical sites like the gluteal region. Application of NPWT following flap procedures yielded improved wound healing outcomes, such as reduced edema and exudate. NPWT also demonstrated efficacy in managing open calvarial wounds caused by osteoradionecrosis, improving flap survival and reconstruction success. When applied to address complex post-surgical wound challenges in sarcoma patients, NPWT contributed to lower complication rates, fewer wound infections, reduced re-interventions, and potential cost savings.

NPWT or VAC is a therapeutic wound dressing technique. The NPWT or VAC system has multiple components. During treatment, a specialized foam or gauze is placed directly on the wound. An adhesive film then seals over the dressing to cover and enclose the wound. A drainage tube connects through an opening in the adhesive film to a portable vacuum pump. The pump applies intermittent or continuous suction to the wound via this tube. This suction effect is thought to aid more rapid wound healing through several mechanisms, such as reduced polymicrobial infections, increased blood flow to the wound site, and removal of static, possibly infectious fluid [28]. While early case reports provided preliminary evidence of NPWT’s safety profile, larger subsequent trials investigated optimized protocols and applications. This review underscores NPWT’s evolving status within OS treatment, as a developing yet still not fully established approach. It highlights the need for additional research to establish optimal protocols, address existing knowledge gaps, and develop clear clinical guidelines for NPWT’s safe and effective implementation [29].

NPWT employs subatmospheric pressure to enhance the treatment of acute, subacute, and chronic wounds [30]. The benefits of NPWT include the effective management and removal of wound secretions; reduction of edema and bacterial load; and stimulation of granulation tissue growth, angiogenesis, localized blood flow, and epithelial migration [31]. These advantages contribute to improved wound healing outcomes and enhanced patient recovery [32].

According to a recent randomized trial in a selected article by PRISMA, NPWT significantly reduced the risk of surgical site infections (SSIs). By integrating this intervention into surgical practice, healthcare providers can mitigate a complication that not only harms patients but also increases healthcare costs. This resulted in a 68.8% reduction in relative risk. Independent findings revealed that SSIs increased hospitalization costs by 23.8% [33]. Similarly, a study aimed to investigate the effectiveness of closed-incision NPWT (ciNPWT) compared to conventional dressings in preventing wound complications following bone tumor resection and reconstruction. The groups were found to have no significant differences in their epidemiological and clinical presentation characteristics. In contrast, the reconstructive options used were significantly different between the two groups. Additionally, group A had lower rates of wound dehiscence, SSI, and need for surgical revision compared to group B. The results support a potential role for this technique in reducing postoperative wound complications and SSIs. A multicenter randomized controlled trial could help provide further clarity around the role and impact of continuous, incisional NPWT after bone tumor excision and reconstruction [34]. Evidently, a recent study (2024) conducted a retrospective review of patients who underwent limb-sparing surgery to remove OS or Ewing sarcoma at a single institution over a seven-year period. The medical records of 39 patients who had limb-sparing resection of femoral bone tumors were analyzed. Data were collected from this group. The outcomes of this investigation revealed that patients who received incisional VAC therapy after surgery had a lower incidence of wound complications compared to those managed with conventional incision dressings (14% vs 50%). Additionally, patients who experienced wound complications had a longer average hospital stay than those without wound issues (five days vs four days). The authors of this work concluded that wound complications have the potential to prolong hospitalization and delay adjuvant chemotherapy treatment for bone tumors. The use of incisional VAC therapy after surgery is associated with a lower risk of wound issues and should be considered for any high-risk surgical wound closure. Reducing wound complications is important as it can minimize disruptions to the treatment plan [35]. In addition, an editorial published in the Journal of Surgical Oncology reported that VAC therapy is an outstanding and adaptable technique that has transformed the field of wound management. Its effectiveness has been extensively demonstrated through considerable documentation, and this study further contributes to the expanding evidence base, highlighting its tremendous postoperative advantages and wide range of uses [36].

In contrast, Diefenbeck et al. suggest via an observational study that VAC therapy does not demonstrate a clear advantage over other treatment options for acute postoperative osteomyelitis in terms of the number of required débridements and recurrence rates after more than three years of follow-up. The outcomes with VAC therapy do not appear to be superior to other established treatment approaches for this condition [37].

Flap Reconstruction Techniques

Flap reconstruction is a surgical technique sometimes utilized by plastic surgeons to close wounds of differing magnitudes. The procedure involves harvesting live, healthy tissue from one area of the body (donor site) and transplanting it to another location (recipient site). Flap reconstruction is used to treat defects involving loss of skin, fat, muscle function, or even bone tissue. During the surgery, the plastic surgeon relocates viable soft tissue or osteocutaneous flaps to reconstruct and close regions impacted by excision or trauma [38].

The outcomes of the present review offer an in-depth analysis of clinical studies over the past 20 years focused on flap reconstructions following extensive OS resections. Several key findings emerge, including decreased recurrence risk, reliable mandible reconstruction, and fewer necessary surgeries. Notably, the vascularized osteoseptocutaneous fibula flap has proved highly effective, offering durable, adaptable tissue-tolerating chemotherapy to facilitate customized implants and successful mandible repairs. Incorporating prosthetics with this approach demonstrated immense value in improving aesthetics, function, and psychological well-being. The studies underscore the importance of interdisciplinary collaboration and meticulous planning, especially for pediatric mandible reconstruction. A noteworthy combined procedure uses an osteocutaneous fibula flap along with a five-component subscapular artery-based flap, consistently achieving good facial defect reconstruction outcomes.

A comprehensive systematic review was conducted to investigate the outcomes of adult lower extremity soft tissue sarcoma excision with plastic surgery flap reconstruction. After a thorough screening process, the mean total follow-up duration was 32.0 ± 24.3 months. Reconstruction involved the use of microvascular free flaps in 65.5% (487/743) of the cases, while the remaining 34.5% (256/743) were local flaps. Postoperatively, 85.8% (307/358) of patients were able to ambulate. Revision surgery was required in 21% of the patients during their respective follow-up periods. The overall limb salvage rate was 93.4% (958/1,026). The pooled surgical outcomes showed that 22.2% (225/1,012) of the patients experienced perioperative complications. The study reveals that while there is room for further optimization of complication rates in the reconstruction of lower extremity soft tissue sarcomas, the multidisciplinary flap reconstructive approach employed in these cases has resulted in high rates of limb salvage and functional postoperative ambulation [39].

In cases where chronic osteomyelitic lesions leave behind significant soft tissue and bone defects after debridement, the use of a local muscle flap can serve as an effective method for closing the wound [40]. By employing this surgical approach along with a specific antimicrobial treatment targeting the responsible microorganisms, the infection was successfully eliminated in 39 of 42 osteomyelitis patients who were monitored for a minimum of two years post-treatment [41]. Similarly, in patients with chronic osteomyelitis, large soft tissue and bone defects can be effectively sealed using local muscle flaps. When these are used in conjunction with comprehensive debridement and targeted antimicrobial treatment, they have been successful in eliminating the infection in 93% of instances [42].

Other Techniques

Surgical treatment of OS continues to advance beyond traditional techniques for large tumor resections. Emerging integrated strategies now combine multiple innovative solutions. For example, some approaches utilize 3D printing and alloy scaffolding to custom-construct personalized prosthetics that offer optimized structures for reconstructing extensive bony defects. Simultaneously, these personalized implants can integrate drug-eluting reservoirs, allowing precise local delivery of chemotherapy directly to the surgery site. In other techniques, specialized extracellular matrices are employed with muscle flaps and tissue-engineered skin grafts to fully reconstruct voids with vascularized, biomechanically appropriate tissues. Even gene therapies are being investigated as adjuvants, aiming to modify the immune response and prevent future metastases. Though still in their early applications, these multimodal solutions hold great promise in improving outcomes for patients with extensive OS involvement. With further refinement, fully individualized treatment protocols may be realized, representing a significant step forward from traditional standards of care. The following section outlines some of these promising techniques.

Occlusive Wound Closure (OWC)

This novel technique describes a novel soft tissue closure method combining three established techniques: skin stretching, NPWT, and delayed primary wound approximation. This synergistic approach is utilized to close large soft tissue defects resulting from tumor excision. Hettwer et al. conducted a study that aimed to evaluate the impact of an alternative wound closure approach after hip tumor arthroplasty compared to standard wound closure using skin staples [43]. This was a single-center, frequency-matched cohort study. The team research reviewed all patients who underwent tumor resection and endoprosthetic reconstruction of the proximal femur due to metastatic bone disease or malignant hematologic bone disease affecting bone at their institution between 2010 and 2014. Those treated with OWC, which involves intradermal suturing, Steri-Strips, and an occlusive skin adhesive, during this period (n = 35) were compared to an equally sized cohort who received standard wound closure with conventional skin staples. Results indicate that OWC may significantly decrease wound complications, antibiotic use, and hospital stay compared to conventional staples in patients undergoing tumor arthroplasty of the hip. As a result, OWC has the potential to help lower the substantially higher risk of prosthetic joint infection seen in this patient group undergoing tumor reconstruction of the hip [43].

Double-Layer Closure (DLC)

Through a study, we aimed to examine the success rates of DLC techniques, specifically the mylohyoideus muscle flap (MMF) for the lower jaw and the pedicled buccal fat flap (BFF) for the upper jaw, in patients with medication-related osteonecrosis of the jaw (MRONJ). The authors conducted a retrospective cohort study enrolling patients diagnosed with MRONJ between 2015 and 2017, who were treated with either the MMF or BFF after removal of necrotic bone areas. Success was assessed as maintaining full mucosal coverage without signs of residual infection at four weeks (T0), four months (T1), and eight months (T3) post-operation. Side effects were also evaluated. Treatment of earlier stage lesions (stages I and II) showed better outcomes than more severe necrosis (stage III). The authors concluded that DLC techniques after MRONJ surgery provide a mechanically stable and well-vascularized structure to cover bone defects. They should be considered as a standard protocol option for all severity levels of the disease [44].

Skin Expansion

Skin stretching is a valuable technique used to facilitate wound closure following large-scale OS removal. This approach involves gradually stretching the skin edges over several days prior to the final closure, in order to reduce the tension on the wound [45]. The skin has viscoelastic and stress relaxation properties that allow it to be gradually stretched and expanded over time. By applying controlled tension on the skin edges, the tissue can be mobilized, and the wound defect can be reduced in size, making primary closure more achievable [46].

The present literature analyzed through this systematic review provided insights into the surgical techniques most commonly used by surgeons to close soft tissue defects after large-scale OS resection. The findings demonstrated that NPWT has emerged as the preferred primary closure method in many cases. NPWT employs subatmospheric pressure to optimize wound bed preparation and promote granulation, enabling definitive closure. At the same time, the review revealed that reconstructive flap procedures constitute another frequently relied upon approach, representing the secondary choice when NPWT is unsuitable or a larger soft tissue void prevents primary approximation. Flap reconstruction harnesses healthy, vascularized tissue to resurface the defect site. Taken together, these results suggest that NPWT and flap reconstruction have become the standard of care among surgeons managing the challenge of large post-resection OS wounds. Further research is needed to refine best practice protocols and continue advancing reconstructive techniques.

## Conclusions

The primary methods for addressing large soft tissue defects following OS resection involve vacuum-assisted wound closure, specialized skin-stretching mechanisms, and an innovative integrated approach combining multiple closure techniques. VAC employs negative pressure to stimulate granulation and shrink the wound bed, preparing it for closure. Skin-stretching devices such as balloon expanders work to gradually enlarge the surrounding skin to reduce tension at the wound edges. This systematic review found that NPWT and flap reconstruction techniques are primarily used by surgeons to close skin and muscle after removing large OSs, as OS often requires extensive resection of the affected bone, leaving a substantial defect. Notably, NPWT uses negative pressure to aid healing, while flap reconstruction covers defects using vascularized tissues, and studies show both effectively support wound closure and recovery following extensive OS removal, enabling patients to rest post-surgery and potentially receive additional adjuvant therapies as needed. Furthermore, this systematic review revealed NPWT to be the most commonly utilized surgical technique by surgeons addressing large soft tissue defects following OS resection. NPWT utilizes sub-atmospheric pressure to promote wound healing and prepare the defect for closure. Reconstructive flap procedures were also frequently employed, representing the second choice, particularly when NPWT was not suitable, or the defect was too extensive for primary closure.

The novel combined methodology synthesizes these options by first using vacuum therapy to prepare the defect site, followed by sequential application of a skin-stretching appliance to widen the borders of available skin for closure. In some cases, additional flap procedures may be incorporated lastly to fully cover any remaining gaps. The overarching goal of these strategies is twofold: first, to promote optimal wound healing through mechanisms like stimulated granulation and reduced tension, and second, to maximize the likelihood of primarily re-approximating the skin without the need for skin grafting. Together, these interventions aim to yield the best postoperative outcomes for patients with large tissue voids following OS neoplasm excision.
